# IL-10 Signaling Blockade Controls Murine West Nile Virus Infection

**DOI:** 10.1371/journal.ppat.1000610

**Published:** 2009-10-09

**Authors:** Fengwei Bai, Terrence Town, Feng Qian, Penghua Wang, Masahito Kamanaka, Tarah M. Connolly, David Gate, Ruth R. Montgomery, Richard A. Flavell, Erol Fikrig

**Affiliations:** 1 Section of Infectious Diseases, Department of Internal Medicine, Yale University School of Medicine, New Haven, Connecticut, United States of America; 2 L2 Diagnostics, LLC, New Haven, Connecticut, United States of America; 3 Department of Immunobiology, Yale University School of Medicine, New Haven, Connecticut, United States of America; 4 Departments of Neurosurgery and Biomedical Sciences, Maxine Dunitz Neurosurgical Institute, Cedars-Sinai Medical Center, Los Angeles, California, United States of America; 5 Department of Medicine, David Geffen School of Medicine, University of California, Los Angeles, California, United States of America; 6 Section of Rheumatology, Department of Internal Medicine, Yale University School of Medicine, New Haven, Connecticut, United States of America; 7 The Howard Hughes Medical Institute, Yale University School of Medicine, New Haven, Connecticut, United States of America; University of Washington, United States of America

## Abstract

West Nile virus (WNV), a mosquito-borne single-stranded RNA flavivirus, can cause significant human morbidity and mortality. Our data show that interleukin-10 (IL-10) is dramatically elevated both *in vitro* and *in vivo* following WNV infection. Consistent with an etiologic role of IL-10 in WNV pathogenesis, we find that WNV infection is markedly diminished in IL-10 deficient (*IL-10^−/−^*) mice, and pharmacologic blockade of IL-10 signaling by IL-10 neutralizing antibody increases survival of WNV-infected mice. Increased production of antiviral cytokines in *IL-10^−/−^* mice is associated with more efficient control of WNV infection. Moreover, CD4^+^ T cells produce copious amounts of IL-10, and may be an important cellular source of IL-10 during WNV infection *in vivo*. In conclusion, IL-10 signaling plays a negative role in immunity against WNV infection, and blockade of IL-10 signaling by genetic or pharmacologic means helps to control viral infection, suggesting a novel anti-WNV therapeutic strategy.

## Author Summary

West Nile virus (WNV), a mosquito-transmitted RNA virus, is a worldwide cause of severe human and animal infection. Mammalian host immune responses to WNV infection are not completely understood and a vaccine or specific therapy is unavailable for use in humans. In the present study, we investigated the putative regulatory role of interleukin-10 (IL-10) during WNV infection in mice. We found that IL-10 signaling facilitates WNV infection and suppresses antiviral cytokine production in response to viral infection. Interestingly, blockade of IL-10 signaling by IL-10 neutralizing antibody increases survival of WNV-infected mice, suggesting a potentially novel therapeutic strategy to combat WNV infection. In addition, we found that CD4^+^ T cells produce a significant amount of IL-10 during WNV infection, providing a more accurate cellular target for IL-10 signaling inhibition. IL-10 also plays a critical role in suppression of excessive inflammation and immunopathology caused by autoimmune diseases or host immune system responses to infections; therefore, safety and efficacy of IL-10 signaling blockade as a therapeutic strategy against WNV infection deserves consideration.

## Introduction

WNV has caused severe morbidity and mortality in both animals and humans since it first appeared in North America in 1999. WNV is maintained in an enzootic cycle between mosquitoes and birds; but humans, horses and other mammals can be inadvertently infected by mosquitoes that carry the virus. Although most human infections are asymptomatic, the elderly and individuals with a compromised immune system are particularly susceptible to life-threatening neurologic disease [1]. Viral pathogenesis is not completely understood, and a vaccine or specific therapy has not yet been approved for use in humans [2]. Experimental mice infected with WNV develop a systemic infection that results in encephalitis and death, thereby mimicking human neuroinvasive disease and providing a valuable model system within which to study viral pathogenesis and immunity.

Mammalian T helper 1 (Th1) cytokine responses are essential for eradicating invading intracellular pathogens. However, if these responses are too strong, bystander effects may damage the host. Interleukin-10 (IL-10), a pleiotropic cytokine, plays a crucial immunosuppressive role during excessive Th1 responses, and can thereby protect the host from potentially damaging immunopathology [3]–[5]. However, excessive IL-10 production suppresses host immune responses and can inadvertently facilitate the ability of intracellular pathogens to escape host innate immune defenses. Epstein-Barr virus (EBV) takes advantage of the immunomodulatory effects of IL-10 by expressing a viral homolog of IL-10 during infections [6]. In addition, recent studies showed that lymphocytic choriomeningitis virus (LCMV) clone 13 elicits high levels of IL-10 production from the infected host, thereby resulting in exhaustion of virus-specific T cells and viral persistence [7]–[9]. Interestingly, neutralization of IL-10 during persistent viral infection leads to recovery of virus-specific T cell responses and reduction of viral load, suggesting a potential therapy to restore T cell function and prevent viral persistence [7],[8].

Type I Interferon (IFN) and Th1 cytokines, including IFN-γ, provide immediate defense against WNV replication and dissemination [10]–[14]. Cellular immunity, including CD4^+^ [15],[16], CD8^+^ T cell [17]–[21] and γδ T cell responses [22]–[24], also participate in host recovery from WNV infection. IL-10 [3] and other regulatory cytokines, such as IL-4 [25]–[28] and IL-13 [29],[30] have been documented to suppress Th1 responses. The role of these regulatory cytokines in modulating Th1 responses during WNV pathogenesis is, however, not completely understood. In the current study, we investigated the role of IL-10 in WNV infection, and our results suggest a promising therapeutic strategy to combat WNV infection through blockade of IL-10 signaling.

## Results

### Expression of *IL-10* is dramatically increased after WNV infection

**Figure 1:**
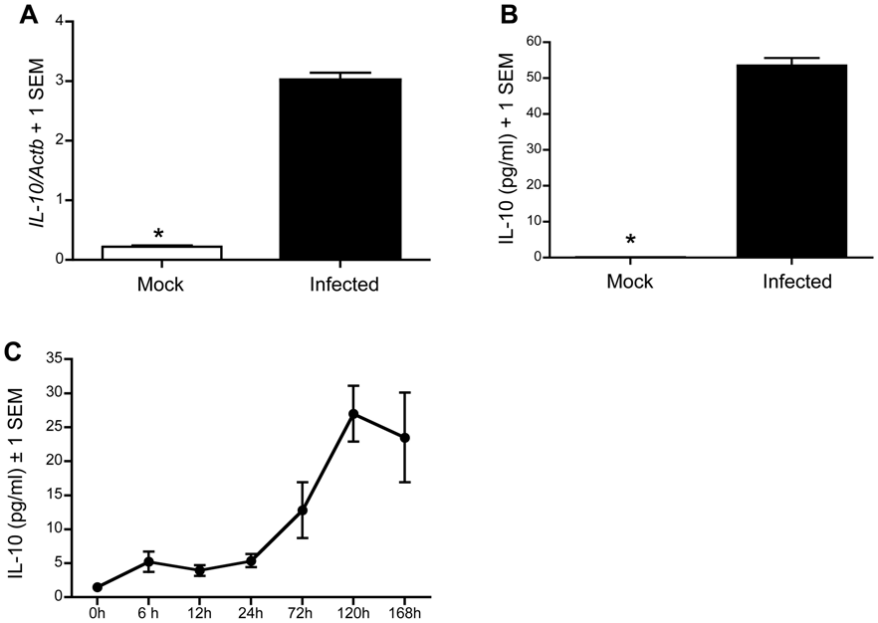
IL-10 expression is elevated *in vitro* and *in vivo* upon WNV infection. Thioglycollate-elicited peritoneal macrophages from C57BL/6 mice were infected with WNV (MOI = 1) for 24 hours. (A), *IL-10* mRNA was measured by Q-PCR and normalized to mouse *β-actin* (*Actb*; unitless ratio+1 SEM). (B), ELISA results are shown for IL-10 protein (pg/ml) in the media. (C), Plasma was prepared at selected time points from C57BL/6 mice infected with WNV (LD_50_), and IL-10 (pg/ml) was measured by ELISA. * *p*<0.001.

To study host immune responses to WNV infection, we performed cytokine expression polymerase chain reaction (PCR) arrays in which cytokine expression profiles were analyzed in thioglycollate-elicited peritoneal macrophages from C57BL/6 mice after WNV infection (multiplicity of infection [MOI] = 1). We observed that *IL-10* expression was up-regulated 4- fold at 5 h and 90-fold at 24 h post-infection (p.i.), while expression of *IL-4*, *IL-13* and another immunosuppressive cytokine, *TGF-β*, were not significantly altered at both time points (data not shown). To confirm this finding, we measured *IL-10* mRNA by reverse transcription quantitative real-time PCR (Q-PCR) and quantified secreted IL-10 protein in cell culture media from primary cultures of WNV-infected macrophages by ELISA. Both Q-PCR and ELISA results confirmed that *IL-10* mRNA and IL-10 protein were markedly up-regulated after WNV infection *in vitro* (**Figure 1 A** and **B**). To further evaluate expression kinetics of *IL-10 in vivo*, we intraperitoneally (i.p.) infected a group of C57BL/6 mice with 2,000 plaque forming units (pfu) of WNV corresponding to a dose at which approximately 50% of wild-type (C57BL/6J) animals survive (LD_50_) [31]–[33] and measured time-dependent IL-10 secretion in plasma by ELISA (**Figure 1 C**). Results showed that IL-10 production began to increase in plasma as early as 6 h p.i. and reached approximately 18-fold at day 5 (120 h) compared to baseline (before infection, 0 h). These data raise the possibility that IL-10 may play a role in modulating immune responses during WNV infection.

### IL-10 signaling facilitates WNV infectivity

**Figure 2:**
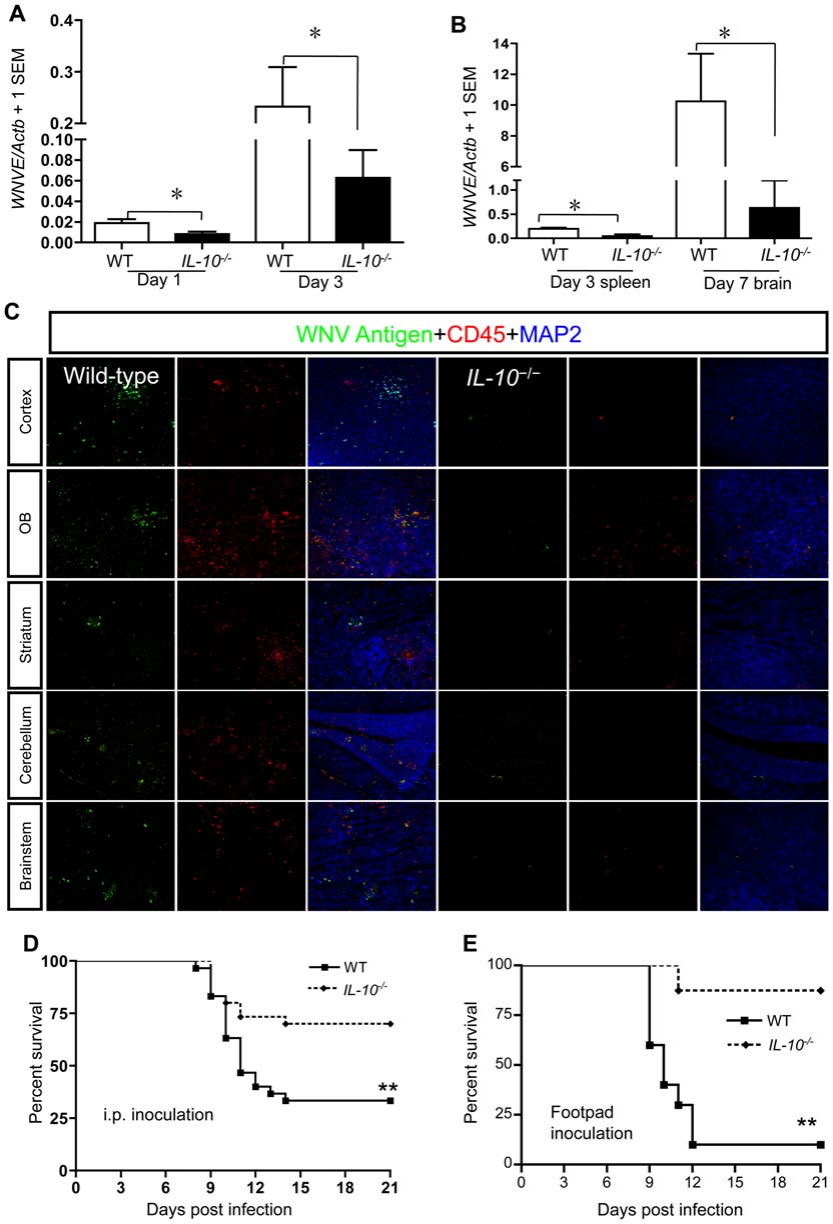
IL-10 signaling facilitates WNV infection. Wild-type (WT, C57BL/6) or *IL-10^−/−^* mice were i.p. challenged with WNV (LD_50_). (A), Q-PCR was performed for *WNVE* in peripheral blood on days 1 (*IL-10^−/−^* mice, n = 14 and WT mice, n = 10) and 3 (*IL-10^−/−^* mice, n = 14 and WT mice, n = 9) p.i.. (B), Q-PCR was performed for *WNVE* in spleens on day 3 (n = 5/group) and brains on day 7 (*IL-10^−/−^* mice, n = 7 and WT mice, n = 9) p.i.. (C), Perfused brains were isolated on day 7 p.i., and WNV antigen (green signal), CD45 (leukocyte common antigen, red signal) and neurons (MAP2, blue signal) were detected by confocal microscopy (OB: Olfactory bulb). These images represent 9 mice per group in 3 independent experiments. (D), Kaplan-Meier survival analysis of *IL-10^−/−^* and WT mice after i.p. inoculation of WNV (n = 30/group). Data shown are pooled from 3 independent experiments. (E), Survival analysis after WNV inoculation by footpad injection (*IL-10^−/−^* mice, n = 8 and WT mice n = 10). Data (means+1 SEM) are pooled results from 2–3 similar independent experiments. *p<0.05 and **p<0.01, compared to control mice.

To evaluate a putative role for IL-10 in WNV pathogenesis, we challenged *IL-10^−/−^* mice with an LD_50_ dose of WNV via an i.p. route of administration [33],[34]. Mice were monitored twice daily for morbidity and mortality for three weeks. Q-PCR designed to measure WNV envelope gene (*WNVE*) revealed 2–3 fold reduced WNV RNA in blood samples from *IL-10^−/−^* mice compared to control mice at days 1 and 3 p.i. (*p*<0.05, **Figure 2 A**). Plaque formation assays were also performed to measure infectious viral particles in plasma samples. Consistent with Q-PCR data, plaque formation assay results showed that *IL-10^−/−^* mice had a similar magnitude of lower viral burden compared with control mice (data not shown). In selected experiments, we randomly sacrificed half the number of mice to collect spleen samples at day 3 and to collect brains at day 7, while the remaining mice were observed for survival analysis. Total RNA extracted from spleen and brain hemispheres was used for Q-PCR analysis of WNV burden. Contralateral brain hemispheres were fixed in 4% paraformaldehyde (PFA) for histological analysis. Markedly less viral RNA was present in the spleens and brains of *IL-10^−/−^* mice *vs.* wild-type mice (*p*<0.05, **Figure 2 B**). Confocal microscopy results were consistent with brain Q-PCR data in that *IL-10^−/−^* mice had little evidence of virus in the olfactory bulb (OB), cerebral cortex, striatum, cerebellum and brainstem, whereas WNV was readily detected in similar brain regions of control mice on day 7 p.i. (**Figure 2 C** and **Figure S1**). Co-immunostaining with the neuronal marker microtubule-associated protein 2 (MAP2) revealed that most of the infected cells were neurons (**Figure 2 C** and **Figure S1**). In addition, a higher number of CD45^+^ leukocytes infiltrated into brains of wild-type mice as compared to *IL-10^−/−^* mice (**Figure 2 C**). Similar results were observed when immunostaining for CD11b, a marker for macrophages/microglia (**Figure S1**). The survival ratio of *IL-10^−/−^* mice (70.0%) was significantly higher than that of wild-type mice (33.3%) (*p*<0.01, **Figure 2 D**). As virus inoculation routes may alter host immune responses, we also challenged *IL-10^−/−^* mice with WNV *via* footpad inoculation (100 pfu) route and performed survival analysis. Similar to intraperitoneal inoculation, *IL-10^−/−^* mice also had increased survival after footpad infection (**Figure 2 E**). Collectively, viral burden and survival analyses demonstrate that mice deficient in *IL-10* have increased resistance to WNV infection, suggesting that IL-10 signaling facilitates WNV pathogenesis.

**Figure 3:**
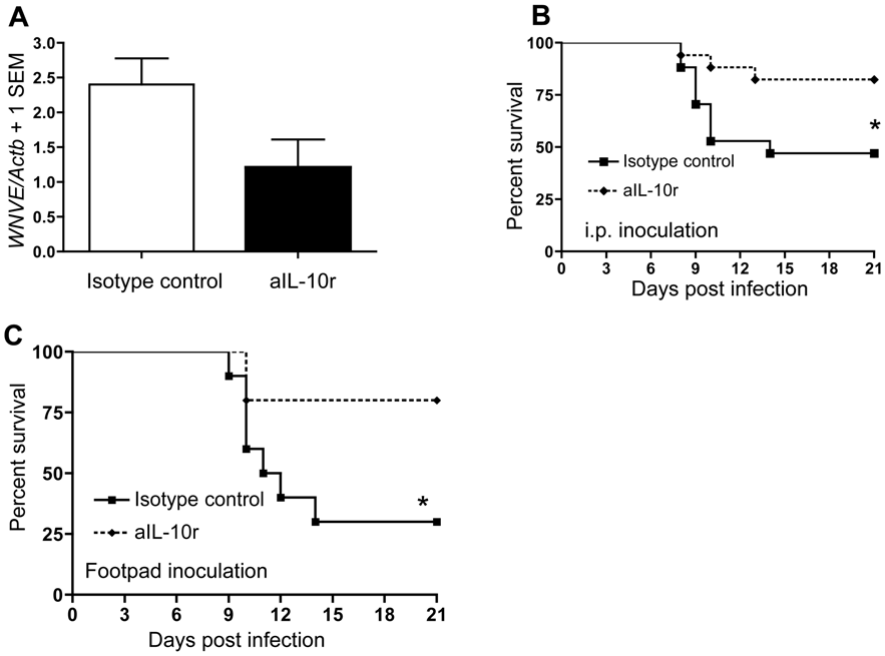
Prophylactic blockade of IL-10 signaling increases survival of WNV-infected mice. A group of C57BL6 mice were i.p. administered anti-IL-10 receptor monoclonal antibody (aIL-10r mAb) or isotype-matched IgG control antibody one day before challenge with WNV. (A), Viral load was measured in blood on day 3 p.i. by Q-PCR (*p* = 0.07, LD_50_, i.p..); (B–C), Kaplan-Meier survival analysis showed significant differences between the aIL-10r mAb and isotype-matched IgG treated group. (B, i.p. inoculation [LD_50_], n = 17/group, data were pooled from 3 independent experiments; C, footpad inoculation [100 pfu], n = 10/group). *p<0.05, compared to control mice.

We further tested this hypothesis by taking a pharmacological approach to interrupt IL-10 signaling. Specifically, we systemically administered anti-IL-10 receptor monoclonal antibody (aIL-10r mAb) to block IL-10 signaling. We injected aIL-10r mAb or isotype-matched control IgG (i.p.) on day 1 prior to WNV infection. Mice were challenged with 2,000 pfu (i.p.) or 100 pfu (footpad inoculation) of WNV. Blood samples were collected from the mice infected intraperitoneally on day 3 (p.i.) for WNV burden Q-PCR measurement. Q-PCR results showed a trend toward reduced viral burden in blood of mice treated with aIL-10r mAb prior to intraperitoneal WNV infection (**Figure 3 A**, *p* = 0.07). Survival analysis indicated that mice treated with aIL-10r mAb had increased survival compared to mice injected with isotype-matched IgG (*p*<0.05, **Figure 3 B** and **C**). These data further confirm that IL-10 signaling facilitates WNV infection and promotion of lethal encephalitis.

### IL-10 signaling suppresses antiviral cytokine production in WNV-challenged immune cells and mice

**Figure 4:**
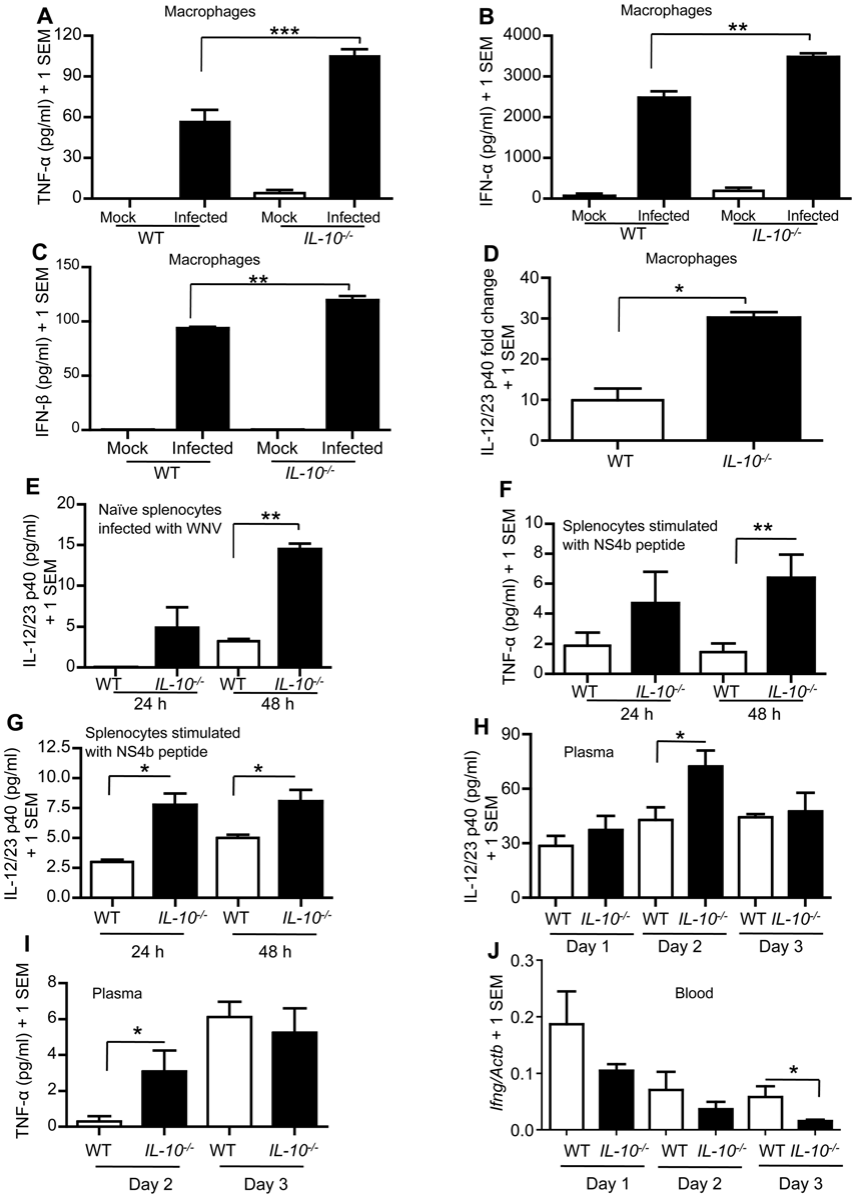
Immune cells and mice deficient in *IL-10* produce more antiviral cytokines. Thioglycollate elicited peritoneal macrophages from *IL-10^−/−^* and wild-type (WT) mice were challenged with WNV (MOI = 1) or PBS (mock infection) for 24 hours. (A–C), TNF-α, IFN-α and IFN-β were measured in media by ELISA. (D), Total RNA from macrophages was analyzed for *IL-12/23 p40* mRNA expression by Q-PCR. The data are normalized to mouse *β-actin* mRNA and are expressed as the relative fold increase over normalized RNA from mock controls. (E), Splenocytes isolated from naïve *IL-10^−/−^*and WT mice were infected with WNV (MOI = 0.5), and IL-12/23 p40 production was measured in media by ELISA. (F and G) Splenocytes isolated from WNV-infected *IL-10^−/−^* or WT mice on day 3 p.i. (LD_50_, i.p.) were stimulated by WNV NS4b peptide (1×10^6^ cells, 1.0 µg/ml) for 24 or 48 hours. TNF-α (F) and IL-12/23 p40 (G) were measured in media by ELISA. (H and I) WT and *IL-10^−/−^* mice were i.p. challenged with WNV (LD_50_) and bled on day 1, day 2 and 3 p.i., and IL-12/23 p40 (H) and TNF-α (I) were measured in plasma by ELISA. (J), Expression of *IFN-γ* (*Ifng*) was measured in peripheral blood by Q-PCR. These data are representative of two independent experiments, with animal numbers ≥4 per group. *p<0.05, **p<0.01 and ***p<0.001.

We have previously shown that macrophages can be infected with WNV, and are likely an important target cell in WNV pathogenesis [33],[34]. To dissect the mechanisms underlying how IL-10 signaling facilitates WNV infection, we measured cytokine expression in macrophages from *IL-10^−/−^* and wild-type mice upon virus infection *in vitro*. We infected thioglycollate-elicited macrophages obtained from peritoneal lavage with WNV, and quantified cytokines by ELISA or Q-PCR at selected time points. Consistent with a stronger anti-viral response, results showed that WNV-infected macrophages from *IL-10^−/−^* mice produced more TNF-α, IFN-α, IFN-β and IL-12/23 p40 than wild-type cells (**Figure 4 A–D**). The spleen is a WNV target organ, and splenic WNV infection is thought to be involved in host immune defense against infection [15],[35]. We therefore assessed cytokine production in splenic cells isolated from naïve and WNV-infected mice. We infected naïve splenocytes with WNV (MOI = 0.5) and measured production of IL-12/23 p40 and IFN-γ by ELISA at selected time points. Splenocytes from *IL-10^−/−^* mice produced elevated IL-12/23 p40 compared to splenocytes from wild-type mice (**Figure 4 E**), while IFN-γ production was not detected by ELISA in either group. We also quantified cytokine production from splenocytes isolated on day 3 p.i.. Specifically, we stimulated splenocytes with WNV MHC class I epitope peptide NS4b [36],[37] *ex vivo* for 24 or 48 h. ELISA results showed that TNF-α and IL-12/23 p40 were significantly elevated in splenocytes from *IL-10^−/−^* mice (**Figure 4 F** and **G**), while IFN-γ production was undetectable. In addition, we evaluated cytokine secretion in peripheral blood of *IL-10^−/−^* compared to wild-type mice during WNV infection (LD_50_, i.p.) by ELISA or Q-PCR. ELISA results showed that IL-12/23 p40 and TNF-α production in plasma from *IL-10^−/−^* mice was higher than wild-type mice plasma samples on day 2 after infection (**Figure 4 H** and **I**). Because IFN protein production in plasma was below the detection level of ELISA, we measured *IFN-γ* mRNA levels in blood by Q-PCR. The Q-PCR data show that expression of *IFN-γ* in blood of *IL-10^−/−^* mice trended toward significantly lower than wild-type mice in all the selected time points, and reached statistical significance on day 3 p.i. (**Figure 4 J**). Collectively, these data suggest that genetic blockade of IL-10 signaling results in a significant elevation of type I IFN and proinflammatory cytokines in macrophages, splenic cells and peripheral blood during WNV infection.

**Figure 5:**
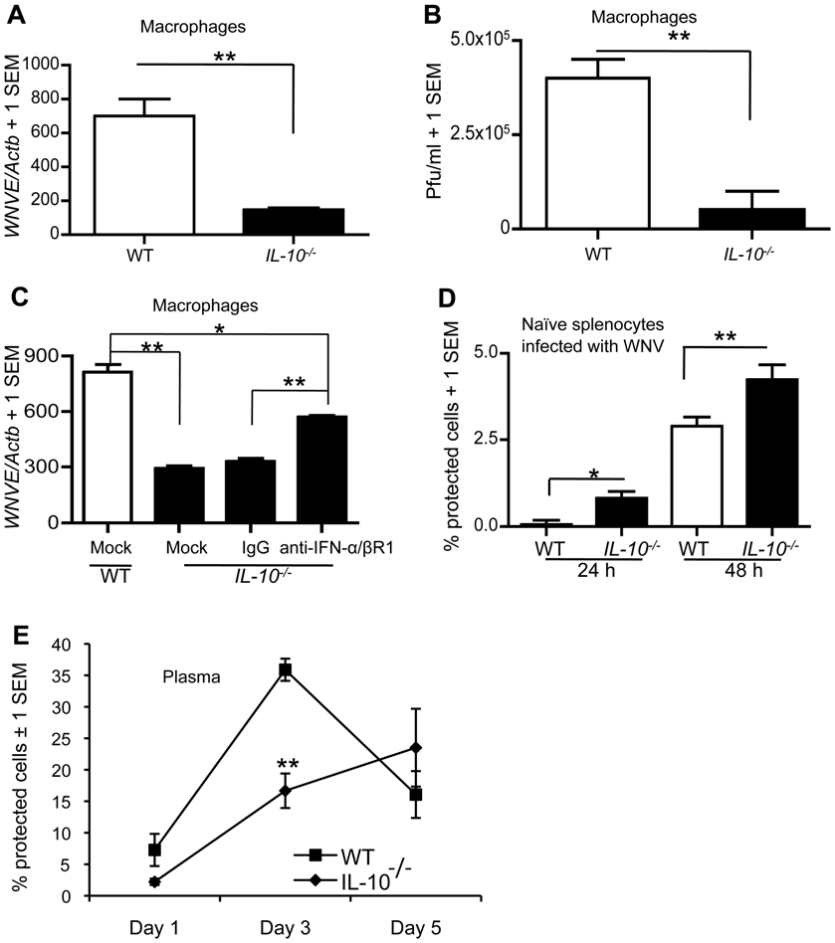
Type I IFN is associated with inhibition of WNV infectivity *in vitro*. (A–B) One million thioglycollate-elicited peritoneal macrophages isolated from *IL-10^−/−^* and WT mice were infected with WNV (MOI = 1) for 24 hours. (A), *WNVE* was measured in macrophages by Q-PCR and (B) viral load was measured in media by plaque formation assay. (C), Macrophages from *IL-10^−/−^* mice were treated with or without (mock) 20 µg/ml of either normal rabbit IgGs or rabbit anti-IFNα/βR1 IgGs (R&D Biosystems) for 2 h followed by WNV infection (MOI = 1) for 24 h. *WNVE* was measured in macrophages by Q-PCR. (D), Splenic cells isolated from naïve WT and *IL-10^−/−^* mice were infected with WNV (MOI = 0.5). Type I IFN level in the medium was measured by IFN bioassay. (E), WT and *IL-10^−/−^* mice were inoculated with WNV (LD_50_, i.p.) and type I IFN level was measured by IFN bioassay. *p<0.05 and **p<0.01.

Increased type I IFN and innate immune proinflammatory cytokines might be expected to contribute to efficient control of WNV infection after IL-10 signaling blockade. To assess this, we evaluated WNV replication in macrophages from *IL-10^−/−^* and wild-type mice *in vitro* by Q-PCR and plaque formation assay. Both assays indicated lower viral burden in macrophages from *IL-10^−/−^ vs.* wild-type mice, showing increased resistance of *IL-10^−/−^* macrophages to direct WNV infection (**Figure 5 A** and **B**). To further assess whether type I IFN plays an antiviral role in macrophages from *IL-10^−/−^* mice, we incubated macrophages with anti-IFN-α/β receptor neutralizing mAb and measured viral replication by Q-PCR after infection with WNV (MOI = 1) (**Figure 5 C**). Q-PCR results showed that neutralizing antibody treatment partially restored WNV replication in macrophages from *IL-10^−/−^* mice. We next measured type I IFN activity in the media of naïve splenic cells that were infected with WNV (MOI = 0.5) using a virus protection assay (murine encephalomyocarditis virus [EMCV] bioassay). These results suggested that splenic cells from *IL-10^−/−^* mice secrete more type I IFN compared to wild-type mice after WNV infection *in vitro* (**Figure 5 D**). The results of the antibody treatment and bioassay experiments indicated that type I IFN production limits viral replication *in vitro* in WNV-infected *IL-10^−/−^* immune cells *in vitro*. To determine if something similar may occur *in vivo*, we measured type I IFN activity in plasma from wild-type and *IL-10^−/−^* mice by EMCV bioassay. Results showed similar type I IFN activity at days 1 and 5 (*p*>0.1), while at day 3, plasma from *IL-10^−/−^* mice had significantly reduced activity compared with plasma from WT mice (*p*<0.01, **Figure 5 E**), which may due to a lower virus burden in the periphery of *IL-10^−/−^* mice.

**Figure 6:**
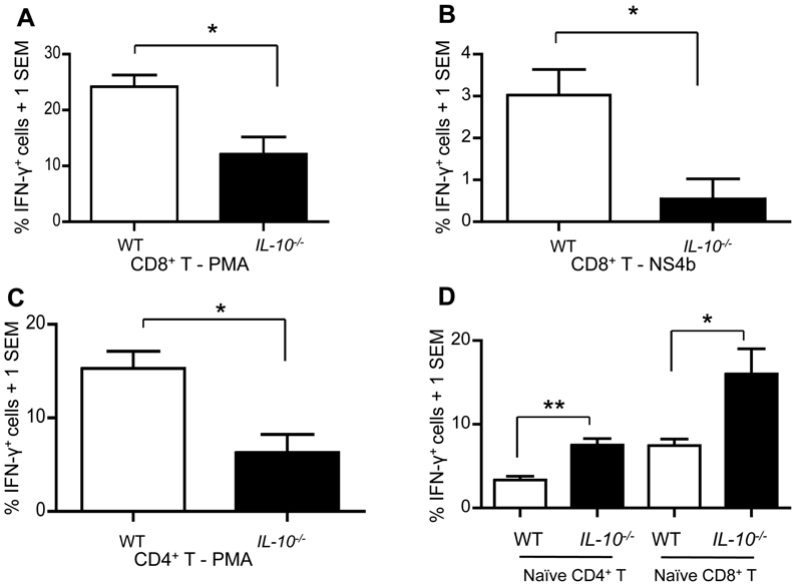
CD4^+^ and CD8^+^ T cells deficient in *IL-10* produce less IFN-γ. (A–C) *IL-10^−/−^* and wild-type (WT) mice were i.p. challenged with WNV (LD_50_). On day 7, mice were euthanized and splenocytes were isolated and stimulated with PMA or WNV NS4b peptide for 6 hours. Flow cytometry analysis of IFN-γ-producing CD8^+^ T cells after stimulation with (A) PMA or (B) with WNV NS4b peptide was then carried out. (C), Flow cytometry analysis of IFN-γ producing CD4^+^ T cells after stimulation with PMA is shown. These data represent two independent experiments (n = 4/group). (D), Splenocytes were isolated from naïve *IL-10^−/−^* and WT mice and stimulated with PMA for 6 hours. Flow cytometry analysis of IFN-γ-producing CD4^+^ and CD8^+^ T cells is shown (n = 3/group). * *p*<0.05 and **p<0.01.

CD4^+^ and CD8^+^ T cells play an important role in recovery of WNV infection in the mouse model [15], [17]–[21]. In addition, host IL-10 expression may result in CD4^+^ and CD8^+^ T cell exhaustion in chronic viral infections [7]–[9]. We investigated whether CD4^+^ and CD8^+^ T cells contribute to increased resistance to WNV infection noted in *IL-10^−/−^* mice. On day 7 p.i., splenocytes were isolated from *IL-10^−/−^* and wild-type control mice and stimulated with PMA or peptide NS4b for 6 h. Total splenic cells were stained for CD4 and CD8 surface markers and for intracellular IFN-γ. Flow cytometry analysis showed that the frequencies of CD8^+^IFN-γ^+^ and CD4^+^IFN-γ^+^ cells were significantly reduced in *IL-10^−/−^* mice (**Figure 6 A–C**). These results are consistent with IFN-γ in blood of *IL-10^−/−^* mice, which trended toward reduced compared with wild-type mice (**Figure 4 J**). These data suggest that IFN-γ does not play a dominant role in mitigation of WNV infection in *IL-10^−/−^* mice. To exclude the possibility that T cells from *IL-10^−/−^* mice may not be as responsive as those that from wild-type mice, we stimulated spenocytes from naïve mice with PMA for 6 h and stained for CD4 and CD8 surface markers and for intracellular IFN-γ. The results show that CD4^+^ and CD8^+^ T cells from naïve *IL-10^−/−^* mice produce more INF-γ than those from wild-type mice (**Figure 6 D**), suggesting that response to PMA stimulation is intact in T cells from *IL-10^−/−^* mice. Collectively, these data suggest that elevated WNV-induced antiviral cytokines in *IL-10^−/−^* immune cells but not IFN-γ production in CD4^+^ and CD8^+^ T cells likely serve to limit viral replication in *IL-10^−/−^* mice.

### IL-10 neutralizing mAb is protective after lethal WNV infection

**Figure 7:**
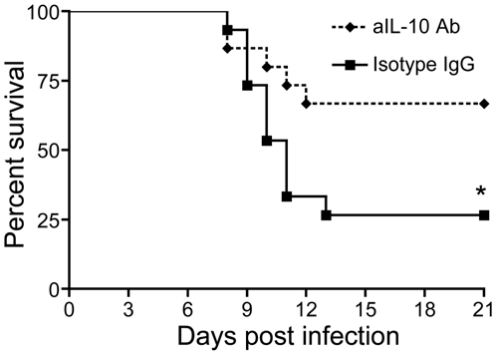
Pharmacologic neutralization of IL-10 increases survival of WNV-infected mice. Wild-type (WT) mice were i.p. challenged with WNV (LD_50_). Mice received two i.p. injections of anti-IL-10 monoclonal antibody (aIL-10 mAb, 200 µg/dose) or isotype control IgG on day 2 and day 3 p.i. (*p*<0.05, n = 15/group). Data were pooled from three independent experiments. * *p*<0.05.

As blockade of IL-10 signaling *via* genetic means leads to enhanced anti-viral immunity mitigating WNV infection, we wanted to assess the effect of pharmacological IL-10 blockade on lethal WNV encephalitis. We used a monoclonal antibody directed against IL-10 (anti-IL-10 mAb) to neutralize IL-10 *in vivo* following WNV infection. Because a treatment course for WNV disease would begin *after* exposure to the virus, we first infected mice with WNV (LD_50_, i.p.) and followed by treatment with two daily i.p. doses of anti-IL-10 mAb or isotype-matched IgG control antibody. Antibodies were injected starting on day 2 or day 4 p.i. Survival curves indicate that anti-IL-10 antibody significantly increases survival of WNV-infected mice when administered as late as day 2 p.i. (*p*<0.05, **Figure 7**). Yet, this effect is abolished when the neutralizing antibody is administrated on day 4 p.i., as survival rates are similar between the two groups (data not shown).

### CD4^+^ T cells produce copious amounts of IL-10 *in vivo* during WNV infection

**Figure 8:**
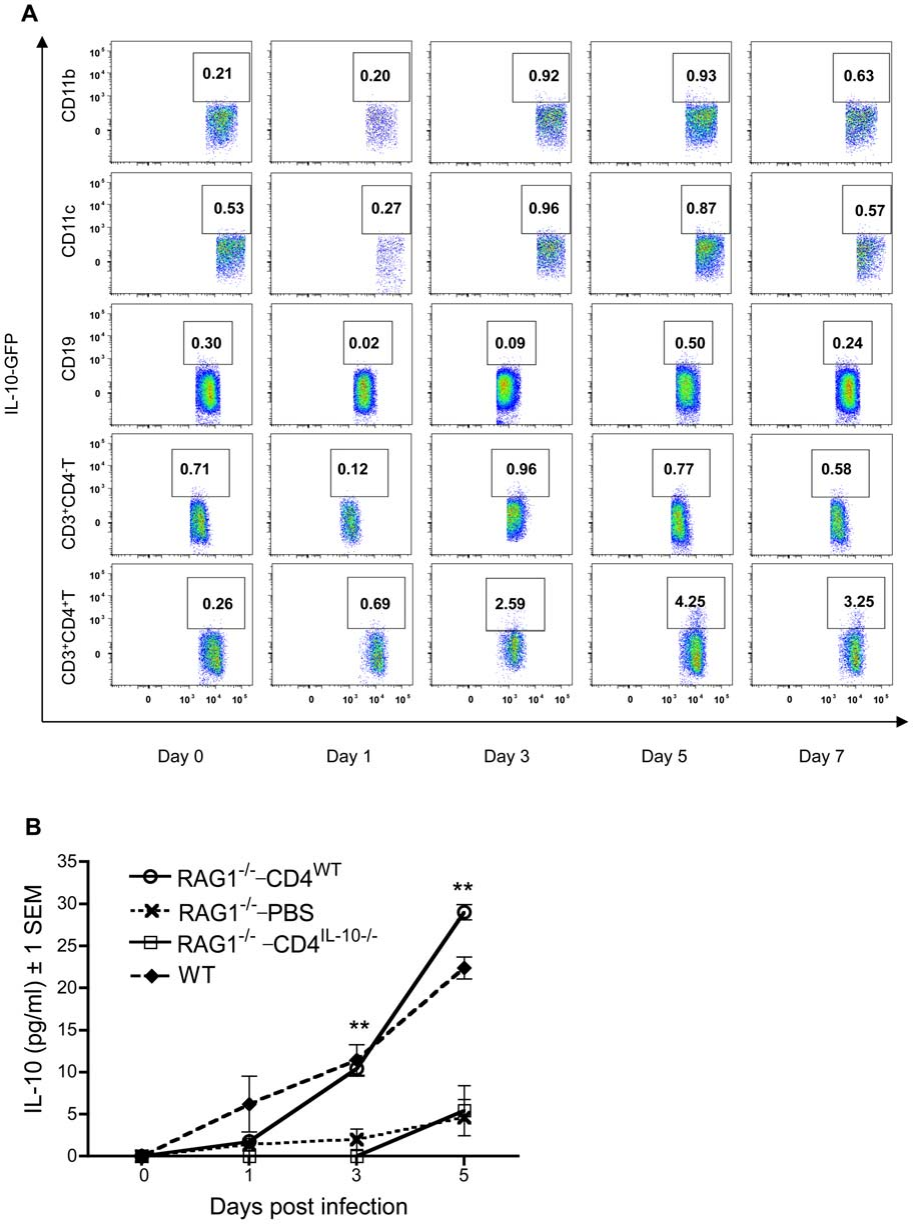
CD4^+^ T cells produce copious amounts of IL-10 *in vivo* during WNV infection. GFP gene knock-in *tiger* and wild-type (WT, C57BL/6) mice were i.p. challenged with WNV (LD_50_). Splenocytes isolated from WNV-infected *tiger* mice at selected time points p.i. were stained for surface markers (day 0, uninfected splenocytes). GFP was used as a surrogate for IL-10 expression, and analyzed by flow cytometry. (A), Percentages of GFP positive cells are shown within CD11b^+^ (macrophages), CD11c^+^ (dendritic cells), CD19^+^ (B cells), non-CD4^+^ (CD3^+^CD4^−^) T cells or CD4^+^ (CD3^+^CD4^+^) T cells populations. Dot-plot figures are representative of at least two independent experiments (n≥2 for each experiment). (B), *RAG1^−/−^* mice were reconstituted with CD4^+^ T cells from naïve WT or *IL-10^−/−^* mice (1×10^7^ cells/mouse) or with PBS as control one day prior to WNV challenge (LD_50_, i.p.). Mice were bled at selected time points and IL-10 production was measured in plasma by ELISA (**p<0.01, *RAG1^−/−^* mice reconstituted with CD4^+^ T cells from WT mice were compared to control mice, n≥3/group).

IL-10 is provided by various cellular sources in different disease models [38]–[40]. A better understanding of the cellular sources of IL-10 and mechanisms of induction is required to define an effective immunotherapeutic strategy aimed at IL-10. To identify the cellular source of IL-10 *in vivo* during WNV infection, we used IL-10-GFP reporter knock-in *tiger* mice, in which an internal ribosome entry site GFP element was inserted into the 3′ region of the IL-10 gene. During characterization of these mice, GFP and IL-10 expression were found to be closely correlated in all cell populations tested [40], thus allowing the identification of IL-10-producing cells *ex vivo* without subsequent manipulation. We examined splenocytes isolated from *tiger* mice for expression of GFP and various cell-surface markers on selected days after WNV infection. No significant GFP production from macrophages (CD11b^+^), dendritic cells (CD11c^+^), B cells (CD19^+^), or non-CD4^+^ T cells (CD3^+^CD4^−^) was observed at any selected time points (**Figure 8 A**). However, a significant amount of GFP was produced by CD4^+^ T cells (CD3^+^CD4^+^), which peaked on day 5 post infection (**Figure 8 A**). This time course of IL-10 induction in CD3^+^CD4^+^ cells is consistent with IL-10 production in plasma of wild-type mice during WNV infection (**Figure 1 C**). These data suggest that CD4^+^ T cells secrete copious amounts of IL-10 during WNV infection in mice.

To further confirm this conclusion, we reconstituted *RAG1^−/−^* mice that lack T and B cells with naïve CD4^+^ T cells from wild-type or *IL-10^−/−^* mice or PBS (cell-free control) on day 1 prior to WNV infection (LD_50_, i.p.). We subsequently measured IL-10 levels in plasma by ELISA at serial time points after infection. Results showed that *RAG1^−/−^* mice reconstituted with CD4^+^ T cells from wild-type mice produced significantly higher amounts of IL-10 than mice reconstituted with PBS or CD4^+^ T cells from *IL-10^−/−^* mice, providing definitive evidence that CD4^+^ T cells produce a significant amount IL-10 during WNV infection in mice (**Figure 8 B**). It is worth noting that IL-10 was also detected in plasma of the *RAG1^−/−^* mice reconstituted with PBS or CD4^+^ T cells from *IL-10^−/−^* mice, albeit at lower levels (**Figure 8 B**), suggesting that cells other than CD4^+^ T cells (such as macrophages) play a certain but subdominant role in secreting IL-10 during WNV infection *in vivo*. Therefore, these data confirm that CD4^+^ T cells produce a significant amount, but not all, of the IL-10 in the murine WNV infection paradigm.

## Discussion

Studies on host immune responses to WNV infection will not only result in a better understanding of viral pathogenesis, but may also lead to novel immunotherapeutic strategies against viral infection. IL-10 plays an immunosuppressive role through interaction between antigen presenting cells and T cells [3],[41],[42]. IL-10 was also found to induce T cell exhaustion during chronic viral infections [7],[8]. Systemic IL-10 production is increased in various human chronic viral infections, such as hepatitis C virus [43],[44], human immunodeficiency virus [45],[46] and hepatitis B virus [47]. Studies demonstrate that the IL-10/IL-10R pathway plays a key role in the establishment of chronic LCMV persistent infection [7],[8]. Blockade of IL-10 signaling converts a chronic LCMV infection into a rapidly controlled acute viral infection and prevents functional exhaustion of memory T cells [7],[8]. Further, a recent study suggests that IL-10 blockade facilitates DNA-vaccine induced T cell response in an LCMV mouse model [48]. Interestingly, IL-10 production correlates directly with plasma HIV viral load and inversely with CD4 cell counts [49].

In this study, we observed that IL-10 expression was dramatically up-regulated *in vitro* and *in vivo* in murine plasma during WNV infection, raising the possibility that IL-10 plays a significant role in modulating host immune responses to WNV infection. Through evaluation of WNV infection in *IL-10^−/−^* mice and in wild-type mice that were administered anti-IL-10r mAb or IL-10 neutralizing antibody to temporally block IL-10 signaling, we found that IL-10 signaling facilitates WNV infectivity and lethal encephalitis. WNV is a neuroinvasive virus that develops low viremia in mice by replicating in peripheral organs including spleen, and then infecting neurons causing meningitis and lethal encephalitis in mice [31],[34],[50]. WNV is most often cleared from blood and peripheral organs between one and two weeks p.i.. In *IL-10^−/−^* mice, WNV load was significantly reduced in the blood and spleen, suggesting that viral replication was efficiently inhibited in the periphery. Consequently, the likelihood that the virus would infect neurons was greatly reduced. We also performed direct intracranial injection of WNV into wild-type *vs. IL-10^−/−^* mice and evaluated survival. We did not detect a difference using this route of infection (data not shown), suggesting that *IL-10* deficiency promotes WNV clearance at the level of the periphery as opposed to the brain.

We next dissected the mechanisms by which IL-10 signaling facilitates WNV infectivity. IL-10 suppresses Th1 responses that are essential to restrict intracellular pathogens [3],[41],[42], and previous studies suggested that IFN and proinflammatory cytokine responses are important in anti-WNV immunity [11], [14], [51]–[53]. Consistent with this notion, we found that these antiviral cytokines were increased in *IL-10^−/−^* macrophages, splenocytes and mouse blood. Macrophages not only produce type I IFN, proinflammatory cytokines and IL-10 upon WNV infection, but they can also be directly infected by WNV. Interestingly, we noted MOI-dependent increases in IL-10 production from wild-type macrophages (MOIs from 0.01 to 1.0), supporting the notion that macrophage IL-10 production is directly correlated with viral abundance. We confirmed that increased abundance of type I IFN and proinflammatory cytokines were associated with inhibition of WNV replication in *IL-10^−/−^* macrophages, and this inhibitory effect was significantly reversed by antagonistic IFN-α/β receptor antibodies. In addition, EMCV bioassay results demonstrated that splenic cells isolated from *IL-10^−/−^* mice produced more type I IFN upon WNV infection. These data are in line with the notion that type I IFN plays an antiviral role in *IL-10^−/−^* immune cells *in vitro*. However, a lower type I IFN activity level in plasma from *IL-10^−/−^* mice compared with wild-type mice at day 3 p.i. was detected by EMCV bioassay (**Figure 5 E**). This may be resolved by the following reasoning. It is possible that higher viral burden in wild-type mice (**Figure 2 A**) stimulates wild-type compared with *IL-10^−/−^* immune cells to produce more type I IFN. This response may be especially evident in the early phase of infection, because at this stage production of regulatory cytokines (such as IL-10) occurs at relatively low levels (**Figure 1 C**).

Studies on infections of bacteria, protozoa and some viruses suggest that expression of *IFN-γ* is up-regulated and participates in controlling infection in the absence of IL-10 signaling [42],[54],[55]. The ability of IL-10 to down-regulate IFN-γ production is a consequence of its ability to inhibit accessory cell function, including production of cytokines (such as TNF-α and IL-12) and expression of costimulatory molecules that are necessary for optimal stimulation of T cells [56]–[59]. In our WNV model, we found that IFN-γ was not inducible in *IL-10^−/−^* immune cells or mice during WNV infection. Studies in chronic infection models have shown that host IL-10 production results in exhaustion of virus-specific T cells and reduction of IFN-γ expression [7]–[9]. We next examined IFN-γ expression on T cells by flow cytometry analysis during WNV infection in *IL-10^−/−^* mice on day 7 p.i.. Results showed that the frequency of IFN-γ-producing CD4^+^ and CD8^+^ T cells was remarkably lower in *IL-10^−/−^* than in wild-type mice (**Figure 6 A–C**). Consistently, IFN-γ expression in blood from *IL-10^−/−^* mice trended toward reduced from day 1 to day 3 p.i. (**Figure 4 J**). In *IL-10^−/−^* mice, viral replication was significantly inhibited following infection (**Figure 2 A** and **B**), indicating that IFN-γ is dispensable for controlling WNV infection in the model of *IL-10^−/−^* mice, particularly during the early stage of infection.

In a previous study, TNF-α was shown to participate WNV control in mouse models [60], and we observed that TNF-α expression was up-regulated in *IL-10^−/−^* mice after WNV infection. It is worth noting that TNF-α has also been shown to increase permeability of the blood-brain-barrier (BBB) during WNV infection [34]. There are a number of variables, including the roles of TNF-α in anti-viral immunity independent of a role in WNV BBB permeabilization, the relative importance of WNV BBB permeability at early *vs.* later stages of infection, and whether WNV entry into the brain via the BBB (as opposed to indirect brain entry via brain penetration of infected leukocytes [34]) is a critical pathway for brain infection and subsequent encephalitis, that are dynamically at play during WNV infection [32], [34], [61]–[63]. These variables make direct determination of the contribution of WNV-induced TNF-α expression in BBB permeability complex and difficult to determine in our *IL-10^−/−^* WNV infection system. We did not examine BBB permeability during WNV infection in the present study because viral replication is significantly inhibited in the periphery of *IL-10^−/−^* mice and the likelihood that WNV would infect neurons is therefore correspondingly low. Thus, it does not appear to be necessary to invoke a BBB permeability hypothesis to explain our current results.

Upon viral infection, host innate immune sensors, including Toll-like receptors, double-stranded RNA-activated protein kinase (PKR), retinoic-acid-inducible protein I (RIG-I) and melanoma-differentiation-associated gene 5 (MDA-5), can be activated to produce type I IFNs and cytokines that limit viral replication and dissemination. In the majority of human infections, WNV is controlled and cleared by anti-viral immune responses before the virus invades the central nervous system. However, for the elderly and immunocompromised patients, for whom innate immune responses are too weak or impaired to efficiently control WNV infection, life-threatening neuroinvasive disease may result. In the present study, we found that IL-10 inhibits host innate immune responses during WNV infection. Importantly, blockade of IL-10 signaling through administration of neutralizing IL-10 antibody was able to partially protect WNV-infected mice from death, suggesting a promising immunotherapeutic strategy. The therapeutic effect of neutralizing IL-10 antibody was significant when administered as late as day 2 p.i., and it is possible that its efficacy may be extended by using higher doses of IL-10 signaling blocking antibodies or small molecule compounds that inhibit the IL-10 signaling pathway. Blocking IL-10 signaling to stimulate host immune responses rather than directly targeting viral genes has the added advantage that it is less likely to lead to the emergence of mutant virus. This anti-WNV strategy may also be combined with other currently available supportive measures, such as IFN therapy, in order to reap better clinical outcomes.

Identification of a major cellular source of IL-10 during WNV infection in mice would, in principle, provide a more accurate target for modulation of IL-10 signaling as a therapeutic strategy. We found that CD4^+^ T cells produce a significant amount of IL-10 and may be a major cellular source of IL-10 *in vivo* during WNV infection. Our data also indicate that macrophages produce IL-10 when exposed to WNV *ex vivo* (**Figure 1 A** and **B**), which is consistent with *in vivo* results showing that CD11b^+^ macrophages produce low levels of IL-10. It is becoming evident that naturally occurring or adaptive CD4^+^ regulatory T cells produce copious amounts of IL-10 in different disease models [38],[39],[42]. Further studies are warranted to identify which CD4^+^ T cell subset is a major IL-10 producing cell during WNV infection *in vivo*. However, it should be noted that such a therapeutic strategy would need to be carefully titrated, as IL-10 signaling can also be beneficial by limiting extensive immunopathological damage resulting from uncontrolled Th1 or lymphokine-responsive T effector cell responses. Thus, while modulation of IL-10 signaling may be a means to enhance anti-pathogen immunity or limit immunopathology, this must be approached with caution to avoid tipping the balance too far in either direction. It is worth noting that we did not observe any overt immunopathology in *IL-10^−/−^* mice during WNV infection, and surviving *IL-10^−/−^* mice appeared active and healthy at the end of our infection experiments. It may be feasible to temporally block or reduce IL-10 signaling in the early phase of acute WNV infection to stimulate the patient's anti-viral immune responses without causing severe immunopathological side-effects. Of course, further pre-clinical studies focused on blocking IL-10 signaling as a therapeutic strategy against WNV infection would need to be explored both in terms of safety and efficacy before moving forward into clinical trials.

## Materials and Methods

### Experimental animals and virus infection

C57BL/6, *IL-10^−/−^*and *RAG1^−/−^* mice were purchased from the Jackson Laboratory (Bar Harbor, ME). IL-10-GFP knock-in *tiger* mice were generated in the laboratory of R. A. Flavell as previously described [40], and were bred as heterozygotes. We performed all experiments on 7–8 week-old mice, and mouse groups were rigorously age- and sex-matched for each infection experiment. West Nile viral isolate 2471 [64] used in these studies was propagated one time in Vero cells and titered in a Vero cell plaque-formation assay. We inoculated mice i.p. with LD_50_ (2,000 pfu) of WNV isolate 2741 in 100 µl of PBS with 5% gelatin. We performed footpad inoculation by injecting 100 pfu of WNV diluted in 50 µl PBS with 1% heat-inactivated fetal bovine serum (FBS) and conducted intracranial inoculation by injecting 20 pfu of WNV in 20 µl PBS with 1% FBS according to a previous report [32]. We observed mice for up to 21 days after infection and checked them twice daily for morbidity (including lethargy, anorexia, and difficulty ambulating) and mortality. All animal experimental protocols were approved by the Yale University Institutional Animal Care & Use Committee. All animal infection experiments were performed in a Bio-safety Level 3 animal facility, according to the regulations of Yale University.

### Cytokine PCR array

One million peritoneal thioglycollate-elicited macrophages were prepared from wild-type or *IL-10^−/−^* mice and challenged with WNV isolate 2741 (MOI = 1) in 6-well culture plates in 2 ml of medium and incubated in a 37°C, 5% CO_2_ incubator. Macrophages were collected at 24 h after washes with PBS for total RNA extraction by Qiagen RNeasy Mini kit (Valencia, CA). The RT^2^ *Profiler™* PCR Array kits were purchased from SuperArray Bioscience Corporation (Frederick, MD), and all assay procedures were conducted in accordance with the kit user manual. Briefly, one microgram of total RNA from *IL-10^−/−^* or wild-type macrophages was transcribed into first strand cDNA and loaded into 96-well PCR array plates with 25 µL Q-PCR master mix per well. After performing Q-PCR, resulting threshold cycle values (C_t_) for all genes were exported into the company-provided data analysis template Excel files for comparison of gene expression between *IL-10^−/−^* and wild-type macrophages. The Q-PCR array experiments were repeated three times with similar results.

### Q-PCR and plaque-formation assay

Total RNA was extracted from macrophages, blood, spleen and brain tissue using the RNeasy kit (Qiagen, Valencia, CA). RNA was used to synthesize cDNA by the AffinityScript Multi Temperature cDNA synthesis kit (Stratagene, La Jolla, CA). WNV-specific RNA was quantified using a Q-PCR technique as we previously reported [65]. Cytokine gene and viral gene copy number were expressed as a ratio to cellular *β-actin* cDNA copies measured by Q-PCR. The ratio of the amount of amplified gene compared with the amount of *β-actin* cDNA represented the relative levels in each sample. Plaque-formation assay procedures were carried out according to our previous report [66].

### Confocal microscopy

Mice were sacrificed under isofluorane anesthesia and transcardially perfused with 60 ml of ice-cold PBS. Brains were rapidly isolated and immersion fixed in 4% PFA in PBS overnight at 4°C, and cryoprotected in a graded series of sucrose (10%, 20%, and 30%, each overnight at 4°C). Para-median sagittal sections were cut at 25 µm using a cryostat. Tissue sections were PAP pen (Zymed Laboratories, South San Francisco, CA) applied and pre-blocked in serum-free protein block (Dakocytomation, Denmark) for 30 min at ambient temperature. Sections were then labeled overnight at 4°C with various combinations of primary antibodies against CD11b (1∶200, Serotec, Raleigh, NC), CD45 (1∶200, Serotec), MAP2 (1∶500, Invitrogen) and/or WNV antigen (from J. F. Anderson; 1∶250). After three rinses in PBS, sections were labeled with appropriate secondary antibodies conjugated with AlexaFluor_488_, -_594_, and/or -_647_ for 1 h at ambient temperature. Following three additional rinses in PBS sections were mounted in fluorescence mounting medium (ProLong Gold, Invitrogen). Images were acquired in independent channels using a Nikon C1 Eclipse laser scanning confocal microscope.

### Interferon bioassay

Type I IFN activity in medium or plasma was detected and quantified using an EMCV bioassay of L929 cells as described [67]. Briefly, serially diluted samples were applied to monolayers of L929 cells (2×10^6^ cells/well) in DMEM containing 10% FBS in 96-well plates. Following incubation for 14 h at 37°C, cells were infected with EMCV. Cells were inspected for cytopathic effects, and 7 h p.i., IFN-mediated protection was assayed using a CellTiter 96 aqueous cell proliferation assay (Promega, Madison, WI). The percentage of protected cells was calculated as described [68], according to the following formula: (optical density at 492 nm [OD_492_] of plasma or supernatant-treated EMCV-infected cells/(OD_492_ of non-EMCV-infected cells − OD_492_ of EMCV-infected cells)/OD_492_ of non-EMCV-infected cells)×100%).

### Cell preparation and culture conditions

Mouse peritoneal macrophages were elicited by i.p. injection with 1 ml of thioglycollate medium (BD BBL™, Franklin Lakes, NJ) and macrophages were collected 3 days later and cultured in DMEM containing 10% FBS and antibiotics. Splenocytes were prepared from naïve or WNV-infected mice at selected time points and cultured in RPMI 1640 medium and infected by WNV (MOI = 0.5) or stimulated with 50 ng/ml of PMA (Sigma-Aldrich, St. Louis, MO) or WNV NS4b peptide (SSVWNTATTAI, 1.0 µg/ml). Cell supernatants were collected for cytokine ELISA analysis using kits purchased from R&D Systems (Minneapolis, MN).

### *In vivo* monoclonal antibody treatment

Seven-week-old female C57BL/6 mice were i.p. injected with 250 µg of mouse anti-IL10r (clone 1B1.3a) or isotype-matched IgG1 (clone R3-34) antibodies in 100 µl of PBS one day prior to WNV infection. Anti-IL10r mAb and isotype control antibodies were purchased from BD Bioscience Pharmingen (San Diego, CA). In anti-IL-10 mAb immunotherapeutic experiments, two doses of 200 µg antibody (clone JES052A5) or isotype control IgG1 (clone 43414, R&D Systems) in 100 µl PBS were injected i.p. daily, with the first dose starting on either day 2 or 4 post-WNV infection.

### Extracellular and intracellular immunostaining

To measure IFN-γ production, splenocytes from WNV-infected mice were isolated and cultured under one of two conditions. Half of the samples were incubated at 3×10^6^ cells/tube at room temperature with no exogenous stimulation for 4 h and Golgi-plug (BD Bioscience Pharmingen, 1.0 µg/ml) was added for the final 2 h (BD Bioscience Pharmingen). We used conditions previously described as optimal for spontaneous accumulation of cytokines after removal from the host [69]. The remaining samples were stimulated at 3×10^6^ cells/tube with 50 ng/ml of PMA (Sigma-Aldrich) or with 1.0 µg/ml of WNV NS4b peptide and 500 ng/ml of ionomycin (Sigma-Aldrich) for 4 h at 37°C and Golgi-plug (1.0 µg/ml) was added during the final 2 h. Cells were then harvested and stained with the following cell surface antibodies (all were obtained from BD Biosciences Pharmingen): CD3 (clone 500A2), CD4 (clone RM4-5), CD8 (clone 53-6.7), CD11b (M1/70), or CD19 (1D3) and fixed in 4% PFA. Cells were then permeabilized with 0.5% saponin before adding FITC-conjugated anti-IFN-γ mAb (BD Bioscience Pharmingen, CA) or isotype-matched control FITC-conjugated rat IgG1 for intracellular staining.

### Splenocyte adoptive transfer

Splenic single cell suspensions were prepared from naïve C57BL/6 or *IL-10^−/−^* mice and CD4^+^ T cells were positively selected using anti-mouse CD4 (L3T4)-conjugated microbeads (Miltenyi Biotec, Auburn, CA) according to the manufacturer's instructions and the purity of eluted cells was assessed by flow cytometry analysis. *RAG1^−/−^* mice received 1×10^7^ CD4^+^ T cells (>98% purity) in 200 µl of PBS per mouse or PBS alone as control.

### Statistical analysis

We calculated standard errors of the means (SEM) and analyzed data by non-paired Student's *t*-test for single mean comparisons. We performed survival curve comparisons using the log-rank test (GraphPad Prism 4.0, La Jolla, CA). *p*<0.05 was considered as statistically significant for all analyses.

### Accession numbers

IL-10 (MGI 96537, PF 00726); WNVE (AF 206518); IFN-α (M 68944); IFN-β (NM 010510); IFN-γ (NM 008337); TNF-α (MGI 104798); IL-12/23 p40 (MGI 96540); Rag1 (MGI 97848); Actb (MGI 87904).

## Supporting Information

Figure S1 Reduced brain WNV infection and CD11b^+^ innate immune cells in *IL-10* ^−/−^ mice. Perfused brains were isolated on day 7 p.i., and WNV antigen (green signal), CD11b (red signal) and neurons (MAP2, blue signal) were detected by confocal microscopy. These images represent 9 mice per group in 3 independent experiments, in which similar results were obtained.
(5.16 MB PDF)
