# Supplementary figures and images for: IL-10 Signaling Blockade Controls Murine West Nile Virus Infection
Source: PLoS Pathog. 2009 Oct 9;5(10):e1000610. doi: 10.1371/journal.ppat.1000610 (PMC2749443; doi:10.1371/journal.ppat.1000610)

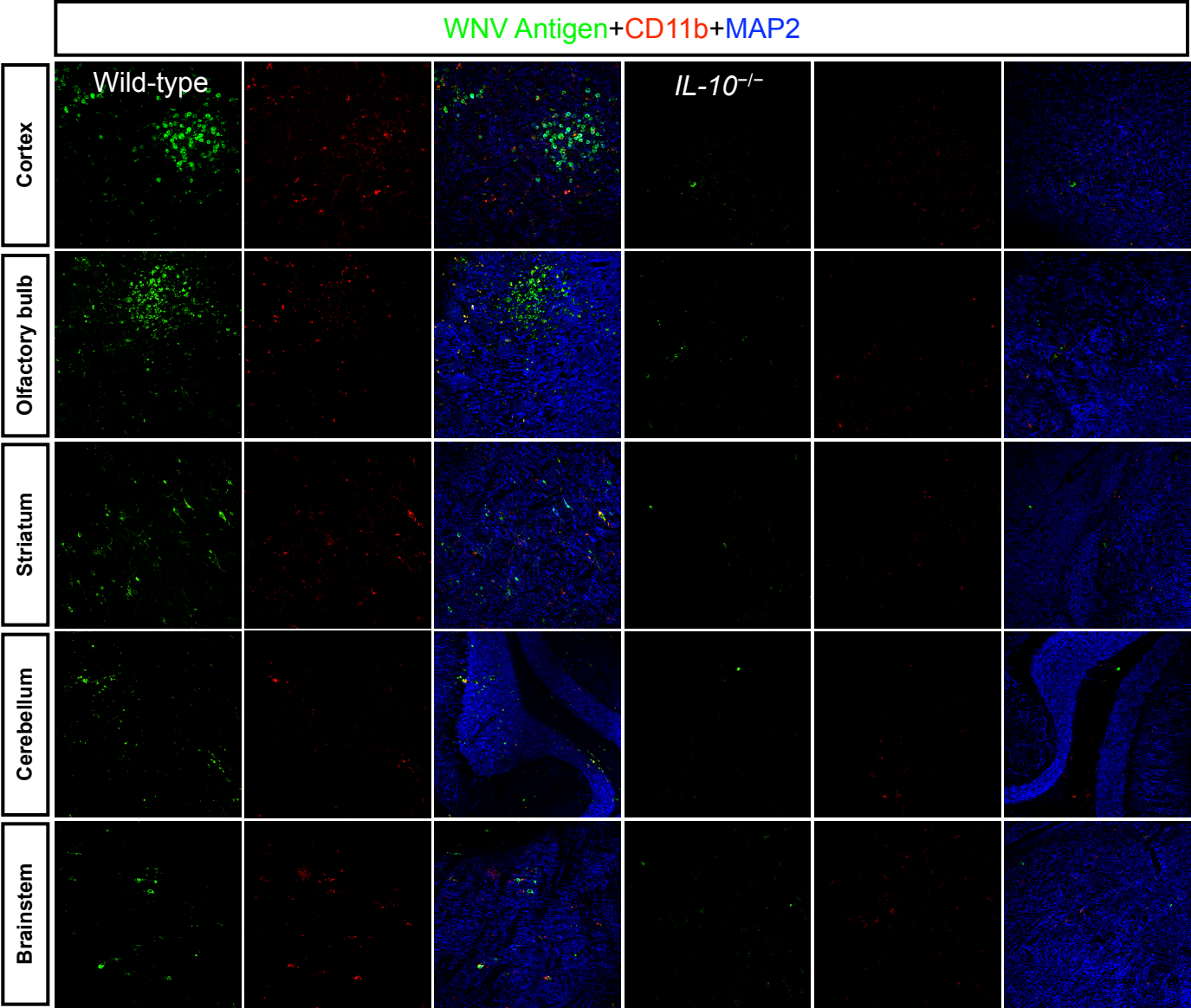

Supplement: Figure S1 — Reduced brain WNV infection and CD11b+ innate immune cells in IL-10 −/− mice. Perfused brains were isolated on day 7 p.i., and WNV antigen (green signal), CD11b (red signal) and neurons (MAP2, blue signal) were detected by confocal microscopy. These images represent 9 mice per group in 3 independent experiments, in which similar results were obtained. (5.16 MB PDF) [file ppat.1000610.s001.pdf]
